# Egyptian Students Open to Digital Mental Health Care: Cross-Sectional Survey

**DOI:** 10.2196/31727

**Published:** 2022-03-21

**Authors:** Mostafa Mamdouh, Andy Man Yeung Tai, Jean Nicolas Westenberg, Farhud Shams, Kerry Jang, Adel Badawy, Houssam Elsawi, Michael Krausz

**Affiliations:** 1 Department of Psychiatry University of British Columbia Vancouver, BC Canada; 2 Department of Psychiatry Tanta University Tanta Egypt

**Keywords:** students, youth, eMental health, Arab countries, mental health care, eHealth solutions, youth mental health, mental health, youth engagement, young adults, EMH, therapy, emotional support, barriers, mobile phone

## Abstract

**Background:**

In Egypt, the shortage of mental health services, particularly for adolescents and young adults, is apparent. Electronic mental health (EMH) has been proposed as a solution to bridge the gap and better address the needs of young people. However, EMH is new to Egypt and its acceptability among target populations is crucial to its implementation and success.

**Objective:**

The objective of this study is to explore the interest of Egyptian youth in EMH, identify perceived barriers to EMH, and inform the design of EMH tools to best address the needs of youth.

**Methods:**

A web-based cross-sectional survey was distributed among medical students at Tanta University in Egypt. Chi-square and one-way analysis of variance tests were performed for inferential analyses using a significance level of .05.

**Results:**

Of the 707 individuals who completed the survey (90.9% response rate), 60.5% (428) were female, 62% (438) lived in urban and suburban areas, and the mean age of the sample was 20.5 (SD 1.8) years. The vast majority of participants (522/707, 73.8%) had already used the internet to find information about mental health problems, but the information was unsatisfactory for about half of them (386/707, 54.6%). Almost all students reported that they would prefer web-based therapy if EMH were available through a trustworthy national web-based platform for youth mental health (601/707, 85%). Students believed that emotional difficulties, social support, and coping strategies were the main topics that EMH should help with. The most common perceived barriers for EMH use in Egypt were concerns about privacy (382/707, 54%) and a lack of technology literacy and unfamiliarity with EMH (352/707, 50%).

**Conclusions:**

EMH is a promising strategy for addressing gaps in the mental health care for young people. To construct and implement a digital system of care that addresses the unique needs and preferences of youth, adolescents and young adults should be involved in the co-development and design.

## Introduction

Globally, mental illness is the most common health problem among postsecondary students [1]. Moreover, without access to appropriate treatment services, mental health problems often worsen throughout an individual’s adult life [2]. It is well established that mental health services are extremely scarce in low- and middle-income countries, estimated to be 200 times lower than that in high-income countries [3]. Youth in low-income countries who struggle with mental illness therefore represent a particularly vulnerable population [1,4]. The aim of this study is to explore electronic mental health (EMH) as a promising strategy to address gaps in the mental health care of young people in the low-income world, by specifically interacting and engaging with students in Egypt.

Though half of the Egyptian population is under the age of 25 years, health care structures are not well equipped to handle mental illness in youth [5]. In a national study of 13,000 high school students in 3 different regions of Egypt, 20%-30% of students had mental health problems, with similar rates reported in several other studies [6]. In Egyptian universities, the prevalence of mental illness is even higher, with up to 37% of undergraduate students fulfilling the criteria for moderate depression [7]. However, there are only 1.44 psychiatrists and 0.11 psychologists per 100,000 people in Egypt [8], and the shortage of mental health services is particularly apparent for youth [5]. Within the government’s mental health workforce, only roughly 3% work in child and adolescent services [9]. Moreover, only around 1% of health care professionals who work in schools are trained in mental health [8].

To provide care, improve access, and build capacity, web-based resources have become increasingly necessary tools, especially during the COVID-19 pandemic [10,11]. EMH interventions have proven effective in providing mental health services to different vulnerable youth populations and have been increasingly used across the world [12]. EMH domains include web-based resources for mental health, such as information, risk assessments, professional and peer counselling, group therapy, cognitive behavioral therapy, and telepsychiatry, that address the plethora of mental illness comorbidities. Implementation of these tools is an iterative process that includes co-development for specific youth populations. Successful EMH platforms for youth mental health services have been developed in different jurisdictions, including Australia, Ireland, and the United Kingdom, with varying designs and methods [12,13]. Such technology-based interventions are also more accessible and cost-effective [14]. According to recent evidence, many Arabs would rather use EMH services than visit a mental health provider, which would help them overcome some preexisting barriers such as stigma, cost, and physical distance [15]. Interestingly, the use of EMH in conjunction with medication-assisted treatment has proven more effective than standalone treatment for substance use disorders in youth [16]. A major strength of web-based interventions is the ability to provide youth with a continuum of care, starting from early diagnosis to continued peer and professional support [17].

EMH is especially relevant to the Egyptian health care system given the high burden of mental health problems, low access to available resources, and high internet accessibility and mobile phone ownership [18]. Existing studies have investigated the implementation and use of platforms such as electronic medical records in Egypt, but no studies have evaluated how youth perceive these technologies [19]. Psychiatrists in Egypt have agreed that EMH could be the solution to building necessary capacity for youth mental health care, especially given the scarcity of resources for this demographic and familiarity with technology among youth [20]. Despite the ubiquity of smartphones and internet access in Egypt and in other Arabic countries, EMH is largely an untapped resource [21,22]. Attempts have already been made using Arabic mobile apps for depression and anxiety, but clear gaps are evident given the low quality, lack of engagement, and absence of evidence-based resources [23]. Crucial to the development and implementation of EMH in Egypt is the opinion of target populations, namely adolescents and young adults. Little is known about the types of mental health information sought by young people on the internet, how EMH can address their needs, and their perceived barriers to using EMH.

The objectives of this study are to gauge the level of interest of Egyptian youth in EMH approaches, highlight features of web-based interventions that are most appealing to students, and identify the perceived barriers to using EMH in this population. Responses were analyzed by gender and living region to best determine the mental health needs of particular target groups among youth. The findings of this study aim to help inform the development and design of EMH tools that best address mental health issues among youth not only in Egypt but also in other Arabic countries. More broadly, this formative research is an integral part of program development, as it explores the feasibility and acceptability of EMH among Egyptian youth before large-scale summative evaluations such as randomized controlled trials.

## Methods

### Survey Design

The survey instrument was initially developed by researchers and health care providers from Tanta University, Tanta, Egypt, and the University of British Columbia, Vancouver, Canada, as part of an ongoing collaboration regarding student mental health. Following its design, 11 students from Tanta University (6 females and 5 males; mean age 20.4 years) were invited to participate in a preliminary analysis of the survey instrument in two 1.5-hour sessions. After revising the instrument based on comments from the students in the workshops, the survey was piloted with a sample of 60 students from Tanta University. Some questions were again modified based on student feedback to improve the overall quality of the instrument. The final version of the instrument was then distributed within the student body of the Faculty of Medicine at Tanta University. Medical school students from Tanta University were selected as the target sample because of their high health literacy level and their ability to address broad issues related to health and well-being and to understand the culture, etiquette, and customs of the general Egyptian public [24].

The survey consisted of 28 questions and included multiple choice questions, dichotomous questions, and Likert scales. The survey was in English since medical education in Egypt is also delivered in English. The survey consisted of both quantitative and qualitative questions meant to explore patterns of internet use and EMH strategies, thereby giving youth a platform to voice their attitudes toward and perceptions of EMH.

### Recruitment

The survey was distributed within the entire student body in October 2019, thereby including students from all 6 years of medical school. Participants were recruited by nonrandom sampling via the student council. The student council of the Faculty of Medicine at Tanta University was provided with a hyperlink to the consent form and questionnaire; the council then sent this link to all students within the student body. When respondents clicked on the link, they were first asked if they were interested in participating in the study. Only those who ticked “yes” could access the survey. Participants were reminded that they could withdraw at any time without giving any justifications and without any negative consequences. Participants were permitted to skip any question they were unwilling to answer and could change any answer over the course of the entire survey. Participation was voluntary and students did not receive any reimbursement.

The following description of EMH was provided to the participants: “E-Mental Health refers to the delivery of mental health services (treatment, information and support) via the internet or mobile phone. This can be through websites, web applications, video conferencing, chat or email. Some of these services, such as video conferencing or online counselling, involve direct one on one contact with a mental health professional. Other e-mental health services, such as web applications or information websites, involve less or no contact with mental health professionals” [25].

### Data Collection and Analysis

Responses were collected electronically using the Qualtrics platform. To eliminate the possibility of duplicated responses, students were allowed to access the survey only once. All data gathered by this study are confidential and anonymous. Chi-square and one-way analysis of variance tests were performed for inferential analyses using a significance level of .05. Descriptive and inferential statistical analyses were executed using SPSS 25 (IBM Corp, 2017) [26]. The results from the web-based survey have been reported according to the Checklist for Reporting Results of Internet E-Surveys [27].

### Ethical Considerations 

The study received approval from the Tanta University Ethics Board (31674/07/17). All participants provided informed consent.

## Results

### Participant Demographics and General Internet Use

The web-based survey link was opened by 778 Egyptian medical school students, of which 707 consented and completed the survey (90.9% response rate). More than half the number of participants were female (428/707, 60.5%), and the mean age was 20.5 (SD 1.8) years (Table 1). All academic years, from first to sixth, were represented in our sample. The majority of participants were living in urban and suburban areas (438/707, 62%), while only 38% (269/707) were living in rural areas (Table 1).

When asked about their internet use, almost all the participants (652/707, 92.2%) said that they used the internet several times a day. Participants mainly accessed the internet using their smartphone (689/707, 97.5%) and mostly from their homes (692/707, 97.8%). The main reasons for using the internet included communication and social media (622/707, 88%) as well as information gathering (611/707, 86.4%; Table 1).

**Table 1:** Participant demographics and internet use (N=707).

Demographics			Value, n (%)
**Gender**			
	Female	428 (60.5)	
	Male	279 (39.5)	
**Age (years)**			
	18	66 (9.3)	
	19	215 (30.4)	
	20	116 (16.4)	
	21	82 (11.6)	
	22	125 (17.7)	
	23	64 (9)	
	24	15 (21)	
	25	24 (3.4)	
**Region**			
	Urban and suburban	438 (62)	
	Rural	269 (38.1)	
**Frequency of internet use**			
	Several times per week	23 (3.3)	
	Once per day	32 (4.5)	
	Several times per day	652 (92.2)	
**Device**			
	Computer	247 (34.9)	
	Smartphone	689 (97.5)	
	Another device	67 (9.5)	
**Place of internet use**			
	Home	602 (97.9)	
	University	351 (49.7)	
	Public areas or transport	306 (43.3)	
	Other	96 (13.6)	
**Main reasons for internet use**			
	Information (content)	611 (86.4)	
	Support (community)	215 (30.4)	
	Communication (social media)	622 (88)	
	Electronic commerce (e-commerce)	52 (7.4)	
	Gaming	276 (39)	
	Others	156 (22)	

### Internet Use for Health Information

The vast majority of participants used the internet to find information about physical and mental health problems (595/707, 84.2% and 522/707, 73.8%, respectively), but the information found on the internet was satisfactory for only about half the number of participants (386/707, 54.6%; Multimedia Appendix 1). Use of the internet to find information about mental health problems was not significantly different between genders and living regions, but significantly more women than men used the internet to find information on physical health problems (373/428, 87.2% vs 222/279, 79.6%; *P*=.007; Multimedia Appendix 1; Table 1). Significantly more individuals from rural areas preferred Arabic to English, in terms of the language of information on the internet (*P*=.001).

**Figure 1:**
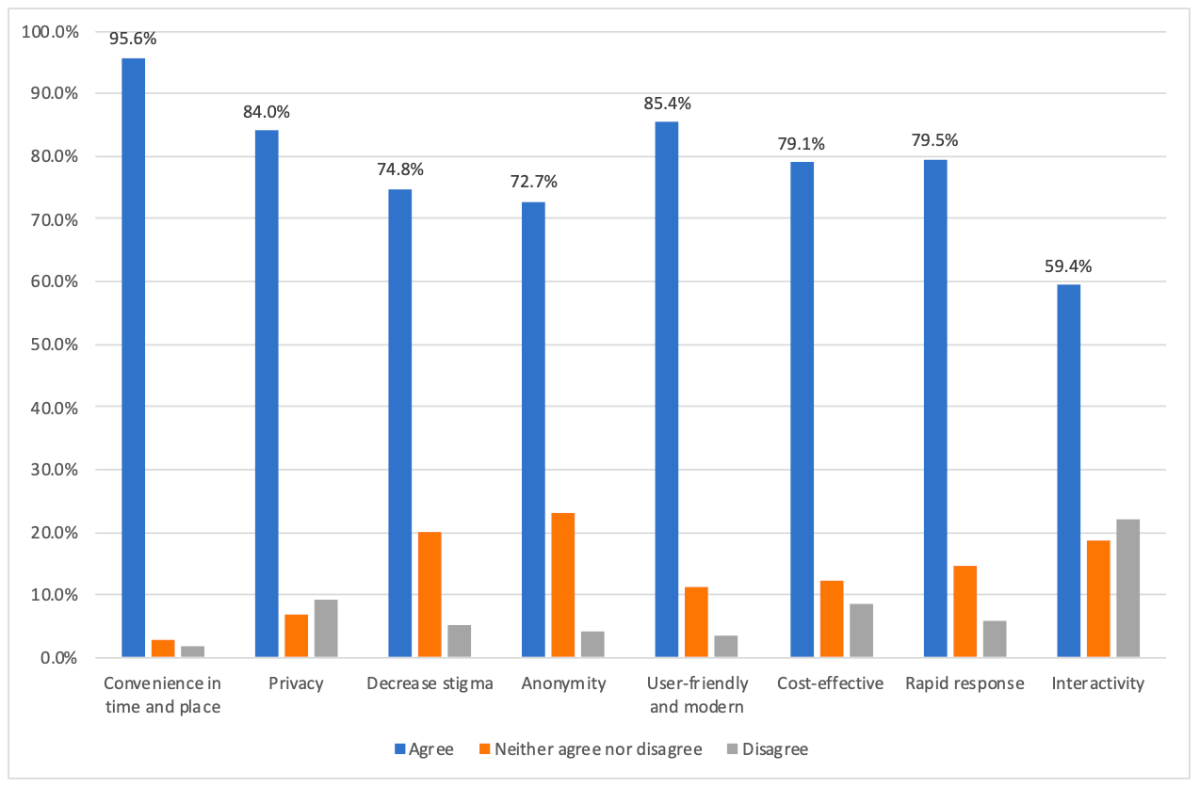
Advantages of web-based interventions. Participants were asked whether they agreed with, disagreed with, or were neutral (neither agreed nor disagreed) toward various advantages of web-based interventions.

Participants revealed that the main reasons for using the internet as a source of web-based mental health help was convenience, user-friendliness, and privacy (696/707, 95.6%, 604/707, 85%, and 594/707, 84%, respectively; Figure 1).

### Knowledge of and Interest in EMH

Over half the number of participants (388/707, 54.9%) did not know that mental health websites and mobile apps existed, and about half the number of participants (359/707, 50.8%) said that web-based mental health services would be an attractive option for them (Table 2). About half the number of participants (366/707, 51.8%) said that they would prefer web-based therapy to conventional psychotherapy. Almost all students (601/707, 85%) reported that they preferred web-based therapy if EMH were available through a trustworthy national web-based platform for youth mental health (Table 2).

**Table 2:** Knowledge of and interest in web-based mental health services (N=707).

Characteristic			Value, n (%)
**Knowledge of preexisting mental health websites and applications**			
	Yes	319 (45.1)	
	No	388 (54.9)	
**Web-based** **mental health services as an attractive option**			
	Yes	359 (50.8)	
	No	107 (15.2)	
	I don’t know	241 (34.1)	
**Preference for** **web** **-based therapy over conventional psychotherapy**			
	Yes	366 (51.8)	
	No	341 (48.2)	
**Preference for web-based therapy if available through a trustworthy national platform**			
	Yes	601 (85)	
	No	106 (15)	

Knowledge about EMH services was significantly different between genders and living regions (*P*<.001 and *P*<.007, respectively; Multimedia Appendix 1; Table 1). Males (149/428, 53.4%) and individuals living in urban areas (215/438, 49.1%) knew more about existing mental health websites and apps than females (170/279; 39.7%) and individuals living in rural areas (104/269, 38.7%), respectively. There were no significant differences in terms of interest in EMH between genders and living regions (Multimedia Appendix 1; Table 1).

### Priorities for and Barriers to EMH Development

Participants believed that emotional difficulties, social support, dealing with stressors, and coping strategies were the main topics that EMH should help with (Table 3). When asked about how participants wanted the information on an EMH platform to be delivered, most participants suggested videos explaining mental health topics (539/707, 76.2%); skills training for improving coping strategies, time management, and self-care (381/707, 53.9%); and web-based mood and behavior assessments (351/707, 49.6%). The most common perceived barriers to EMH use were concerns about confidentiality and privacy issues (382/707, 54%), uncertainty toward and unfamiliarity with EMH (352/707, 50%), and technical difficulties (242/707, 34%).

**Table 3:** Priorities for and barriers to electronic mental health development (N=707).

Characteristic			Value, n (%)
**Priorities for EMH^a^**			
	Emotional difficulties	431 (60.1)	
	Social support	424 (60)	
	Dealing with stressors	420 (59.4)	
	Learning coping strategies	403 (57)	
	Mental health	365 (51.6)	
	Sexual education	240 (34)	
	Physical well-being	230 (32.5)	
	Self-harm behaviors	218 (30.8)	
	Cultural- and religious-sensitive topics	207 (29.3)	
	Substance use	148 (21)	
	Others	52 (7.4)	
**Information displayed on EMH**			
	Videos to help explain mental health topics	539 (76.2)	
	Skills training modules	381 (53.9)	
	Web-based tools to assess mood and behavior	351 (49.7)	
	Self-guided web-based intervention	312 (44.1)	
	Pictures to help explain mental health topics	298 (42.2)	
	Family involvement and support	248 (35.1)	
	Web-based peer connection	167 (23.6)	
	Information delivered in game format	147 (20.8)	
	Other	40 (5.7)	
**Barriers to EMH use**			
	Privacy issues and confidentiality	382 (54)	
	Technical issues and difficulties	242 (34.2)	
	Cost	166 (23.5)	
	Validity and reliability	191 (27)	
	Uncertainty toward or unfamiliarity with EMH	352 (49.8)	

^a^EMH: electronic mental health.

There were many significant differences between genders and living regions (Multimedia Appendix 2; Table 2). More women thought that web-based platforms should help in dealing with stressors (*P*=.03) and self-harm behaviors (*P*=0.03), whereas more men were interested in seeing web-based platforms help with coping strategies (*P*<.001), sexual education (*P*<.001), and substance use (*P*=.02). Individuals living in urban areas reported coping strategies (*P*=.01), sexual education (*P*=.001), and cultural- and religious-sensitive topics (*P*=.003) as priorities.

## Discussion

### EMH

Among Egyptian university students between 18 and 25 years of age, the vast majority report using the internet mainly for social media and general information gathering, which are findings similar to those of previously reported surveys in the same age group from different countries such as the United States, England, and Spain [28-30]. Moreover, Egyptian youth use the internet to find information about mental health, which was also seen among European students [31]. The internet is certainly a popular source of information for youth around the world and seems to be an accessible medium for mental health information.

In Egypt, there is high demand for EMH services that are safe and reliable, and which may be well addressed by a nationally supported platform. A notable example of such a trustworthy and nationally implemented EMH program is eheadspace in Australia, which provides web-based and phone support services to vulnerable youth [32]. eheadspace includes 3 different tools, including group chats, web-based interactive dashboards, and private chats. Group chats increase communication and better enable web-based discussions facilitated by mental health professionals between students and like-minded individuals. The dashboard allows vulnerable youth to collect and manage resources that they find helpful for their daily routines [32]. Private chat sessions accompanied by health professionals can provide youth with intimate conversations that help meet their own individual needs [33]. Since its launch in 2012, eheadspace has created a fully operational eMental health digital ecosystem with an eMental health portal that provides funded services to Australian youth. eheadspace has proven to be an effective solution and has been a key part of the infrastructure addressing mental health among Australian youth [34]. Countries in the Middle East could follow suit to create effective and much needed EMH platforms across the region.

Web-based therapy relative to conventional therapy would be preferred for many Egyptian students if they were diagnosed as having mental illness. This is consistent with findings of an Iranian study showing that students were more willing to use EMH services if available [35]. Conversely, a majority of Irish university students reported preferring face-to-face support rather than web-based support for a mental health problem because it was more reliable and allowed better communication [25]. This discrepancy between youth of different countries, mainly European and Middle Eastern countries, is likely due to several different factors such as stigma around mental health and accessibility to in-person mental health services. Indeed, the scarcity of face-to-face services and resources in Egypt could push students toward web-based solutions if those are most available to them. Students in Egypt reported accessibility and convenience among the main reasons for preferring EMH as a solution for mental health support along with other important benefits such as anonymity and mitigating stigma. More generally, individuals with mental health issues tend to be less resistant to the use of EMH programs if these are effective, and they report willingness to pursue services if these are made easily accessible to them [36].

### Differences in EMH Needs

As expected, women and men reported different perceived needs and opinions regarding web-based services. For instance, women seemed more interested than men in searching the internet for physical health problems possibly because of a higher prevalence of stigma experienced by women with mental illness that leads to the somatization of psychological symptoms, which is common in Arab countries [37]. In terms of priorities for EMH, women expressed more interest in interventions that could help address self-harm behaviors and that could help deal with stressors. Conversely, men seemed to want EMH interventions to provide education on coping behaviors, sexual education, and substance use. Moreover, individuals living in rural and urban areas also reported varying needs. For instance, rural populations seemed to prefer the language to be Arabic on the internet, whereas urban populations preferred English. This highlights the importance of considering language in the provision of web-based resources. The different needs of the various subpopulations demonstrate the importance of providing mental health information that is as individualized as possible. In general, it seems that the relationship between patients and physicians has become less hierarchical and more client-provider oriented [38]. EMH solutions can address a wide variety of issues in this regard and offer functionalities to tailor content to the needs of the individual, for example, by gauging a user’s need through in-app assessments [39].

### Barriers to EMH

The most important perceived barrier for Egyptian students was confidentiality and privacy of personal information. This is in agreement with the results of a web-based survey that examined consumer expectations and potential challenges of EMH services across several countries such as Australia, Iran, the Philippines, and South Africa [35]. Interestingly, the highest rates of participant willingness to try EMH services are seen in low-income countries such as Iran and Egypt, which also reported a high number of barriers and concerns with patient confidentiality and the protection of personal information [35]. It is important for program developers to recognize the functional and technical assistance that individuals may need to use such services [35]. In high-income countries, there seems to be less concern with how data are handled, possibly because of past positive experiences in dealing with sensitive information on already developed web-based health platforms [40]. Another common concern among Egyptian students was their unfamiliarity with the range of functionalities in EMH as well as technical approaches. These findings were similar to those of another cross-sectional study of university students in the United Arab Emirates, in which half the number of participants had never heard of mobile mental health care apps and 75% had never used these kinds of apps even during the COVID-19 pandemic [41]. Considering that the survey sample consisted of medical school students, familiarity is likely even lower in the general young population and thus the barriers to EMH use could be greater. These findings highlight the need for increased discussions around the use of EMH in the Middle East. EMH should be a topic in the curriculum for all Middle Eastern students to increase their comfort with it, thereby furthering its implementation and acceptability. Increasing the presence of EMH resources in the everyday lives of youth would also allow for better co-design and collaboration between developers and students. This would help address concerns on the validity and reliability of the available content, which was also highlighted as a perceived barrier by the youth in our sample [42].

### Limitations

The findings are not completely generalizable to the general young population because the sample comprised well-educated and technologically savvy students. Moreover, the questionnaire was web-based, and all participants had to use the internet to complete the questionnaire, which demonstrated at least some degree of digital literacy. It must also be considered that all students at this particular university are expected to use the internet as a way of staying up to date on courses. Future studies should examine perceptions about EMH in other subpopulations in Egypt, such as in university students from other specialties (not health care related) and youth not enrolled in postsecondary educational programs.

### Conclusions and Implications

The findings of this study highlight that young people attending university are active users of the internet and are willing to use the internet for mental health information and support. EMH is therefore a feasible strategy for addressing gaps in the mental health care for young people in Egypt. Moreover, target populations (eg, males vs females, urban vs rural) as well as topics of interests (eg, self-harm, substance use) must be considered when implementing web-based solutions, given the different needs and preferences of these populations as outlined by this study. Finally, EMH platforms should prioritize informational videos and skills training modules as a way to display content while also addressing privacy and confidentiality issues, which were identified as barriers to EMH use among Egyptian students.

Based on the results of this survey, a virtual mental health clinic is being developed for the students at Tanta University, which will be the first web-based and evidence-based intervention designed specifically for university students in Egypt. The clinic, which will be developed in collaboration with international and local experts, will provide support to students and begin to address gaps in the mental health care for young people in Egypt. Nevertheless, further studies in this area are needed to better understand the feasibility of EMH in the broader Egyptian population and in other Arabic countries. Researchers and mental health clinicians in the Middle East must work together with the youth in their countries to develop and implement web-based interventions that are accepted and used by this population. Students should strive to develop and use EMH platforms to provide compassion and love for the generations to come.

## Appendix

Multimedia Appendix 1 Knowledge of and interest in electronic mental health services by gender and region.

Multimedia Appendix 2 Perceived priorities of electronic mental health among Egyptian students by gender and region.

## Abbreviations

EMH: electronic mental health
