# Supplementary material for: Egyptian Students Open to Digital Mental Health Care: Cross-Sectional Survey
Source: JMIR Form Res. 2022 Mar 21;6(3):e31727. doi: 10.2196/31727 (PMC8981018; doi:10.2196/31727)
Supplement: Multimedia Appendix 1 [file formative_v6i3e31727_app1.docx]

Multimedia Appendix 1. Knowledge of and interest for online mental health by gender and region.

|  | | Male | Female | *p*-value | Urban | Rural | *p*-value | Total |
| --- | --- | --- | --- | --- | --- | --- | --- | --- |
| Using the internet to find information about physical health problems; n (%) | |  |  |  |  |  |  |  |
|  | Yes | 222 (79.6) | 373 (87.2) | 0.007* | 366 (83.6) | 229 (85.1) | 0.579 | 595 (84.2) |
|  | No | 57 (20.4) | 55 (12.9) |  | 72 (16.4) | 40 (14.9) |  | 112 (15.8) |
| Using the internet to find information about mental health problems; n (%) | |  |  |  |  |  |  |  |
|  | Yes | 200 (71.7) | 322 (75.2) | 0.294 | 320 (73.1) | 202 (75.1) | 0.55 | 522 (73.8) |
|  | No | 79 (28.3) | 106 (24.8) |  | 118 (26.9) | 67 (24.9) |  | 185 (26.2) |
| Satisfaction with information found on the internet; n (%) | |  |  |  |  |  |  |  |
|  | Dissatisfied | 22 (7.0) | 57 (13.3) | 0.08 | 48 (11.0) | 31 (11.5) | 0.079 | 79 (11.2) |
|  | Neither satisfied nor dissatisfied | 98 (35.1) | 114 (33.60 |  | 137 (31.3) | 105 (39.0) |  | 242 (34.2) |
|  | Satisfied | 159 (57.0) | 227 (53.0) |  | 253 (57.8) | 133 (49.4) |  | 386 (54.6) |
| Preferred language for information found; n (%) | |  |  |  |  |  |  |  |
|  | Arabic | 121 (43.4) | 165 (38.6) | 0.202 | 157 (35.8) | 129 (48.0) | 0.001* | 286 (40.5) |
|  | English | 138 (56.6) | 263 (61.5) |  | 281 (64.2) | 140 (52.0) |  | 421 (59.6) |
| Knowledge of pre-existing mental health websites and applications; n (%) | |  |  |  |  |  |  |  |
|  | Yes | 149 (53.4) | 170 (39.7) | <0.001* | 215 (49.1) | 104 (38.7) | 0.007* | 319 (45.1) |
|  | No | 130 (46.6) | 258 (60.3) |  | 223 (50.9) | 165 (61.3) |  | 388 (54.9) |
| Online mental health services as an attractive option; n (%) | |  |  |  |  |  |  |  |
|  | Yes | 130 (46.6) | 229 (53.5) | 0.093 | 228 (52.1) | 131 (48.7) | 0.392 | 359 (50.8) |
|  | No | 51 (18.3) | 56 (13.1) |  | 69 (15.8) | 38 (14.1) |  | 107 (15.1) |
|  | I don't know | 98 (35.1) | 143 (33.4) |  | 141 (32.2) | 100 (37.2) |  | 241 (34.1) |
