# Supplementary material for: Egyptian Students Open to Digital Mental Health Care: Cross-Sectional Survey
Source: JMIR Form Res. 2022 Mar 21;6(3):e31727. doi: 10.2196/31727 (PMC8981018; doi:10.2196/31727)
Supplement: Multimedia Appendix 2 [file formative_v6i3e31727_app2.docx]

Multimedia Appendix 2. Perceived priorities of EMH among Egyptian students, by gender and region.

| What do you think the online platform should help with?; n (%) | | Male | Female | *p*-value | Urban | Rural | *p*-value |
| --- | --- | --- | --- | --- | --- | --- | --- |
|  | Learning coping strategies | 185 (66.31) | 218 (50.93) | <0.001* | 266 (60.73) | 137 (50.93) | 0.011* |
|  | Sexual education | 133 (47.67) | 107 (25.00) | <0.001* | 169 (38.58) | 71 (26.39) | 0.001* |
|  | Social support | 168 (60.22) | 256 (59.81) | 0.915 | 257 (58.68) | 167 (62.08) | 0.37 |
|  | Dealing with stressors | 152 (54.48) | 268 (62.62) | 0.031* | 261 (59.59) | 159 (59.11) | 0.899 |
|  | Substance use | 71 (25.45) | 77 (17.99) | 0.017* | 96 (21.92) | 52 (19.33) | 0.412 |
|  | Mental health | 151 (54.12) | 214 (50.00) | 0.284 | 227 (51.83) | 138 (51.30) | 0.892 |
|  | Culturally and religiously sensitive topics | 76 (27.24) | 131 (30.61) | 0.336 | 146 (33.33) | 61 (22.68) | 0.003* |
|  | Physical well-being | 97 (34.77) | 133 (31.07) | 0.306 | 135 (30.82) | 95 (35.32) | 0.216 |
|  | Emotional difficulties | 165 (59.14) | 266 (62.15) | 0.423 | 262 (59.82) | 169 (62.83) | 0.426 |
|  | Self-harm behaviors | 73 (26.16) | 145 (33.88) | 0.030* | 125 (28.54) | 93 (34.57) | 0.092 |
|  | Others | 8 (2.87) | 44 (10.28) | <0.001* | 36 (8.22) | 16 (5.95) | 0.261 |
